# Optical, Photophysical, and Electroemission Characterization of Blue Emissive Polymers as Active Layer for OLEDs

**DOI:** 10.3390/nano14201623

**Published:** 2024-10-10

**Authors:** Despoina Tselekidou, Kyparisis Papadopoulos, Konstantinos C. Andrikopoulos, Aikaterini K. Andreopoulou, Joannis K. Kallitsis, Stergios Logothetidis, Argiris Laskarakis, Maria Gioti

**Affiliations:** 1 Nanotechnology Laboratory LTFN, Department of Physics, Aristotle University of Thessaloniki, 54124 Thessaloniki, Greece; kypapado@physics.auth.gr (K.P.); logot@auth.gr (S.L.); alask@physics.auth.gr (A.L.); 2 Department of Chemistry, University of Patras, Caratheodory 1, University Campus, 26504 Patras, Greece; k.andrikopoulos@ac.upatras.gr (K.C.A.); andreopo@upatras.gr (A.K.A.); kallitsi@upatras.gr (J.K.K.); 3 Organic Electronic Technologies P.C. (OET), 20th KM Thessaloniki—Tagarades, 57001 Thermi, Greece

**Keywords:** blue-light emitting OLEDs, polyfluorene, polycarbazole, spectroscopic ellipsometry, color selectivity

## Abstract

Polymers containing π-conjugated segments are a diverse group of large molecules with semiconducting and emissive properties, with strong potential for use as active layers in Organic Light-Emitting Diodes (OLEDs). Stable blue-emitting materials, which are utilized as emissive layers in solution-processed OLED devices, are essential for their commercialization. Achieving balanced charge injection is challenging due to the wide bandgap between the HOMO and LUMO energy levels. This study examines the optical and photophysical characteristics of blue-emitting polymers to contribute to the understanding of the fundamental mechanisms of color purity and its stability during the operation of OLED devices. The investigated materials are a novel synthesized lab scale polymer, namely poly[(2,7-di(p-acetoxystyryl)-9-(2-ethylhexyl)-9H-carbazole-4,4′-diphenylsulfone)-co-poly(2,6-diphenylpyrydine-4,4′-diphenylsulfone] (CzCop), as well as three commercially supplied materials, namely Poly(9,9-di-n-octylfluorenyl-2,7-diyl) (PFO), poly[9,9-bis(2′-ethylhexyl) fluorene-2,7-diyl] (PBEHF), and poly (9,9-n-dihexyl-2,7-fluorene-alt-9-phenyl-3,6-carbazole) (F6PC). The materials were compared to evaluate their properties using Spectroscopic Ellipsometry, Photoluminescence, and Atomic Force Microscopy (AFM). Additionally, the electrical characteristics of the OLED devices were investigated, as well as the stability of the electroluminescence emission spectrum during the device’s operation. Finally, the determined optical properties, combined with their photo- and electro-emission characteristics, provided significant insights into the color stability and selectivity of each material.

## 1. Introduction

To date, electrically semiconducting polymers have proved to be irreplaceable materials in the development of various electronic and optical devices, such as Organic Light-Emitting Diodes (OLEDs), Organic Field Effect Transistors (OFETs), Organic Photovoltaics (OPVs) etc. Among the main advantages are their solubility in common solvents, mechanical flexibility, non-expensive fabrication, and processing, with conductivity levels comparable to those of inorganic semiconductors or even metals [1,2,3,4,5,6]. Therefore, understanding their basic properties is essential for the design of novel semiconducting polymers, which are used as emissive layers and applied in solution-processed OLED devices. Wet-based deposition techniques are the most attractive methods for achieving flexible large-area full-color displays at a low cost due to their compatibility with roll-to-roll fabrication [7,8,9,10]. In particular, the emergence of flexible and wearable electronic devices as a part of the Internet of Things could be an important driving force to a new commercialization area of these materials, semiconducting polymers capable of responding adequately to these devices’ requirements [11].

Notably, there is a significant need for research on improved conjugated polymers towards the three primary colors. It is well known that OLED devices require red, green, and blue emissions with high stability, efficiency, and color purity. It is important to mention that red and green light-emitting materials have been exhibiting excellent luminous efficiency and spectral stability [3,12,13,14,15,16]. On the other hand, developing blue light-emitting materials presents a significant challenge, leading to increased research interest in producing stable and high-quality blue light [17,18]. The intrinsic wide band gaps of blue emissive materials result in a high charge injection barrier and unbalanced injection and transportation of charges [15].

Specifically, the conjugated polymers containing the Poly-fluorene (PF) and Poly-carbazole (PCz) motifs are promising candidates for such blue-light emitting materials. These materials have garnered increased attention owing to their good electro- and photoactive properties, as well as their high hole transporting mobility and strong absorption in the UV spectral region [11]. These materials also provide good thin film morphology and, for this reason, are promising candidates for application as an emissive layer in OLED devices [8,14,16]. In addition, they can be utilized as host materials for internal color conversion in blends with other conjugated polymers and with phosphorescent dyes providing easy color tuning [14,19,20,21,22].

However, fluorene-based derivatives may suffer from poor color purity and stability. Under prolonged device operation or annealing of the materials in air, PF-type materials often appear to degrade, resulting in long-range emission at photon ranges of 2.2–2.3 eV. There are two possible mechanisms that have been proposed and intensively debated to explain the origin of the undesirable emission band centered at 540–550 nm: (i) excimer emission due to interchain aggregation, or (ii) fluorenone formation in 9-position due to oxidation. For the first possible mechanism, initially, reordering of the polymer chains and subsequent aggregation, as well as excimer formation, was assigned as the source of the green emission. Instead of that, the second possible phenomenon was associated with on-chain defects incorporated during synthesis. Their oxidation leads to the presence of ketone defects, yielding the so-called fluorenone moieties incorporated into the polymer backbone. Currently, it is widely agreed that ketone defects are responsible for green emissions. As a result, the color of the emission shifts from the desired blue to the blue-green region (or even yellow). In order to improve the performance of PF-based OLED devices, it is important to identify the origin of the red-shift emission and to understand the mechanism of color degradation [6,23,24,25,26,27,28,29].

To realize improved performance, it is essential to synthesize new blue-light emitting materials with high color stability and selectivity for blue OLEDs. Significant efforts have been made to design versatile blue fluorescent materials aiming at further improving device efficiency, chromaticity, and lifetime. To this end, the combination of the synthesis of novel blue-emitting polymers and simple wet-fabricated OLEDs remains an open issue in the research field of OLEDs.

In this work, we present the comparative study of the optical and photophysical properties of a new synthesized lab-scale polyethersulfone, namely poly[(2,7-di (p-acetoxystyryl)-9-(2-ethylhexyl)-9H-carbazole-4,4′-diphenylsulfone)-co-poly(2,6-diphenylpyrydine-4,4′-diphenylsulfone] (CzCop), with three commercially supplied blue-emitting polymers, the poly(9,9-di-n-octylfluorenyl-2,7-diyl) (PFO), the poly[9,9-bis(2′-ethylhexyl) fluorene-2,7-diyl] (PBEHF), and the poly (9,9 n-dihexyl-2,7-fluorene-alt-9-phenyl-3,6 carbazole) (F6PC). These emissive polymers consist mainly of fluorene and carbazole units. Their derivatives are based on alternating the fluorene and carbazole units or modifying the main chain with side groups in order to achieve better solubility in common solvents and film-forming ability. The commercially available fully conjugated blue-emitting polymers are directly compared with CzCop, which, apart from the carbazole moiety, also differentiates itself in that it incorporates a polyether motif in the main chain, disrupting the conjugation of the emitting moieties with oxygen heteroatoms. Specifically, aromatic polyethersulfones have been investigated as potential polymeric materials to be used in the emissive layer of OLEDs, incorporating fluorescent or even phosphorescent moieties, displaying attractive properties in terms of their facile synthesis, easier purification, film-forming ability, and ease of processability [30,31]. These materials could be applied in solution-processed OLED devices as emissive layers. The fabricated OLEDs have been subsequently studied and characterized in terms of their electroluminescence properties. This work aims to fully define and compare the innovative lab scale polymer with the commercially available ones, focusing on the selective stable blue emission. Determining the materials’ optical properties in combination with the devices’ photo- and electro-emission characteristics provides us with the necessary information to investigate and discuss the possibilities for applying these polymers to the proposed blue OLED devices, and the first encouraging results are obtained.

## 2. Materials and Methods

### 2.1. Materials

Copolymer CzCop were synthesized according to previously published procedures described elsewhere [27]. Poly(9,9-di-n-octylfluorenyl-2,7-diyl), PFO (Mw = 114,050) was supplied by Ossila (Sheffield, UK), whereas poly[9,9-bis(2′-ethylhexyl) fluorene-2,7-diyl], PBEHF (Mw = 79,000) and poly (9,9 n-dihexyl-2,7-fluorene-alt-9-phenyl-3,6 carbazole) F6PC (Mw = 9195) were supplied by Sigma Aldrich Chemie GmbH (Taufkirchen, Germany).

### 2.2. Ink Formulation

**Scheme 1:**
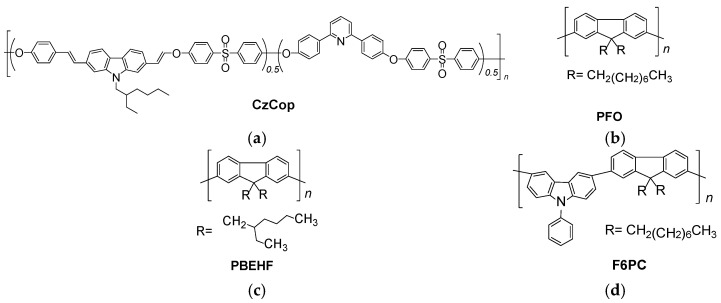
Chemical structure of emitting polymer: (**a**) CzCop, (**b**) PFO, (**c**) PBEHF, and (**d**) F6PC.

For the Hole Transport Layer (HTL), a solution of poly-3,4-ethylene dioxythiophene: poly-styrene sulfonate (PEDOT:PSS, Clevios Heraus Germany, Leverkusen, Germany) AI 4083 mixed with ethanol in the ratio of 2:1 was prepared. The PFO, PBEHF, and F6PC polymers were dissolved in chloroform with a resulting concentration of 1% wt. The synthesized copolymer CzCop (Mw = 69,000) was dissolved in N,N-Dimethylformamide (DMF) with a consequent concentration of 1% wt. The chemical structures for all studied polymers are depicted in Scheme 1.

### 2.3. OLED Fabrication

**Scheme 2:**
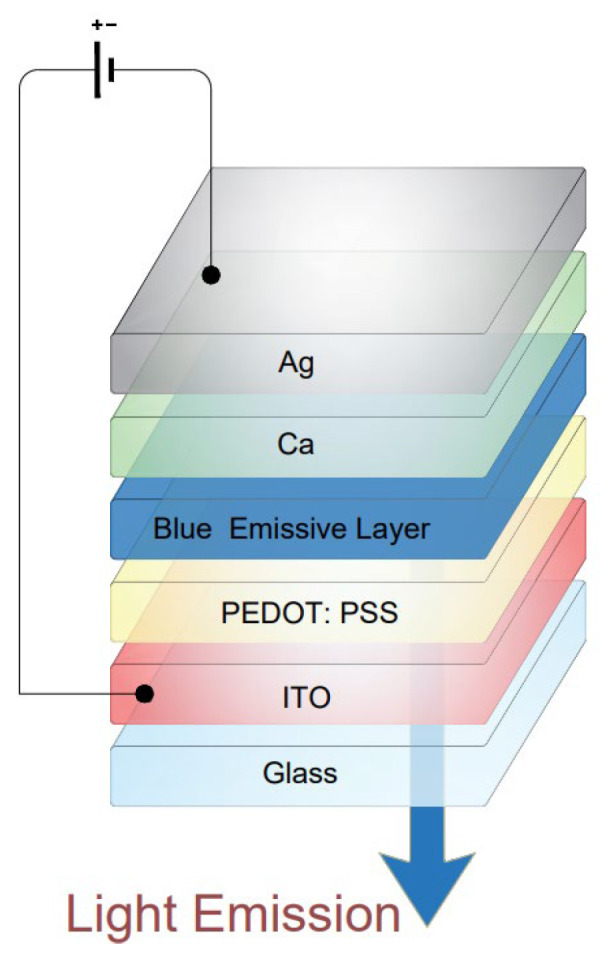
The architecture of the fabricated devices.

The fabricated OLED devices are structured as shown in Scheme 2. Firstly, pre-patterned Indium-Tin Oxide-coated (ITO) glass substrates (received by Ossila, Sheffield, UK) were extensively cleaned by sonication in DI, acetone, and ethanol for 10 min, followed by drying under nitrogen. The substrates were also treated with oxygen plasma at 40 W for 3 min. Then, the PEDOT:PSS layer, which was used as the HTL, was deposited by the spin coating method onto the glass/ITO substrate, followed by annealing at 120 °C for 5 min. The emitting layers (EML) were spun at the same speed, 2000 rpm/s for 60 s, onto the PEDOT:PSS layer. Finally, a bilayer of Ca with a thickness of 6 nm and Ag with a thickness of 125 nm, which was used as an electron transport layer and cathode, respectively, was deposited using the appropriate shadow masks by Vacuum Thermal Evaporation (VTE).

### 2.4. Thin Film and Device Characterization

Spectroscopic Ellipsometry (SE) is a powerful and robust, non-destructive, and surface-sensitive optical technique for the determination of the optical properties as well as the thickness of the light-emitting polymers. Through the SE technique, we can measure the pseudodielectric function of the studied thin films. Moreover, by employing suitable modeling and fitting procedures, we can obtain valuable information regarding the dielectric function *ε*(*ω*), the precise thickness of the nanometer-scale thin films, the absorption coefficient, and optical constants such as the fundamental band gap and absorption energies (optical gaps). The SE measurements were conducted using a phase-modulated ellipsometer (UVISEL JobinYvon, Horiba Europe Research Center, Palaiseau, France) from the near IR to far UV spectral region 1.5–6.5 eV with a step of 20 meV at 70° angle of incidence. The SE experimental data were fitted to model-generated data using the Levenberg–Marquardt algorithm, which took into consideration all the fitting parameters of the applied model.

(1) $$\langle \varepsilon \left(\omega \right)\rangle =\left\langle \varepsilon_{1}\left.\left(\omega \right)\right\rangle \right.+i\left\langle \varepsilon_{2}\left.\left(\omega \right)\right\rangle \right.$$

The surface morphology of the emitting thin films was investigated by Atomic Force Microscopy (AFM) (NTEGRA, NT-MDT, Moscow, Russia) in ambient conditions, using the tapping scanning mode and silicon-based cantilevers with a high-accuracy conical tip and nominal tip roundness < 10 nm.

Finally, the Photoluminescence (PL) and Electroluminescence (EL) characteristics of the active layers and of the final OLED devices, respectively, were measured using the Hamamatsu Absolute PL Quantum Yield measurement system (C9920-02, Jokocho, Higashi-ku, Hamamatsu City, 431-3196, Japan) and the external quantum efficiency system (C9920-12, Jokocho, Higashi-ku, Hamamatsu City, 431-3196, Japan), which measures brightness and light distribution of the devices. The current density-voltage and the luminance-voltage characteristics of the devices were measured using the Electroluminescence technique.

## 3. Results

### 3.1. Spectroscopic Ellipsometry

The optical and electronic properties of the blue-light emitting polymers were determined by modeling and analyzing the measured complex pseudo-dielectric function, 〈*ε*(*Ε*)〉, via SE in the visible to far ultraviolet (Vis-fUV) spectral region. To obtain quantitative information from the measured 〈*ε*(*Ε*)〉 spectra, this has been analyzed by the use of a 5-phase theoretical model consisting of the layer sequence air/blue-light emitting polymer/PEDOT:PSS/ITO/Glass. We sequentially measured each layer and used the appropriate theoretical optical model to calculate the optical constants and thickness of each layer.

**Figure 1:**
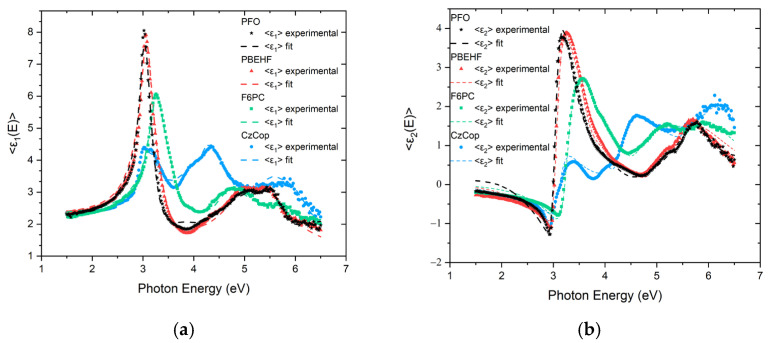
The experimental (symbols) (**a**) real $\left\langle \varepsilon_{1}\right.\left(\left.Ε)\right\rangle \right.$ and (**b**) imaginary $\left\langle \varepsilon_{2}\right.\left(\left.Ε)\right\rangle \right.$ spectra of the pseudodielectric function and the corresponding fitted ones (lines) of the studied films, grown on Glass/ITO/PEDOT:PSS.

Figure 1a,b show the experimentally measured real part $\left\langle \varepsilon_{1}\right.\left(\left.Ε)\right\rangle \right.$ and the imaginary part $\left\langle \varepsilon_{2}\right.\left(\left.Ε)\right\rangle \right.$ (symbols) spectra of the pseudo-dielectric function 〈*ε*(*Ε*)〉, as a function of the photon energy in the range of 1.5–6.5 eV, as well as the corresponding fitted ones (dash lines). Specifically, for the determination of the dielectric response of the emitting polymers PFO, PBEHF, F6PC, and CzCop, we have used the modified Tauc–Lorentz (TL) dispersion oscillator model, which has been successfully applied in amorphous organic semiconductors [32,33,34,35]. The TL model is a powerful tool that can accurately describe interband absorptions above the energy bandgap and is presented in detail in previous works [32,36]. The imaginary part of the dielectric function, which is directly related to electronic absorption, is given by the expressions:

(2) $$\varepsilon_{2}\left(E\right)=\sum_{i}\frac{A_{i}E_{0i}{\Gamma_{i}\left(E-E_{g}^{TL}\right)}^{2}}{{\left(E^{2}-E_{0i}^{2}\right)}^{2}+\Gamma_{i}^{2}E^{2}}\cdot \frac{1}{E},\;E>E_{g}^{TL}$$

(3) $$\varepsilon_{2}(E)=0,\;E\leq E_{g}^{TL}$$

where $E_{g}^{TL}$ is the energy band gap, $E_{0i}$ the resonance energy, $\Gamma_{i}$ the broadening, and $A_{i}$ the strength of the ith oscillator.

**Figure 2:**
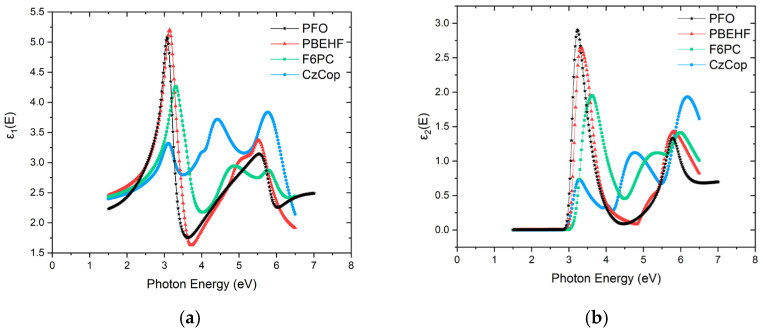
The (**a**) real *ε*_1_(E) and (**b**) imaginary *ε*_2_(E) part of the dielectric function *ε*(*Ε*) of the studied films were calculated using the best-fit parameters derived by the SE analysis procedure.

Figure 2a,b show the calculated real $\left\langle \varepsilon_{1}\right.\left(\left.Ε)\right\rangle \right.$ and imaginary $\left\langle \varepsilon_{2}\right.\left(\left.Ε)\right\rangle \right.$ parts of the dielectric function of the studied polymers derived from the best-fit analysis. Specifically, we used three (*i* = 3) TL oscillators for the commercially available polymers PFO and PBEHF, four (*i* = 4) and five (*i* = 5) TL oscillators for the F6PC and the synthesized CzCop, respectively, to accurately parameterize all the electronic transitions. The ε(E) is determined based on the best-fit parameters from the analysis of the measured $\left\langle \varepsilon \right.\left(\left.Ε)\right\rangle \right.$. This analysis provides insights into the thickness of the thin film, the energy of characteristic electronic transitions, and the energy band gap of the materials under investigation. The respective calculated values of these parameters are presented in Table 1.

The dielectric response of blue-emitting materials is examined in relation to their electronic transition energies. For all studied polymers, the first electronic transition energy is calculated to be approximately E_01_ ≈ 3 eV. However, the differences are evident and more pronounced at the higher energies range, above 3.5 eV. The PFO and PBEHF exhibit similarities in E_04_ and E_05_ energy values, which are significantly weaker in comparison to the E_01_. The F6PC shows similar strength, but the E_02_ and E_03_ are calculated in lower energies. Finally, in the case of CzCop, it is a reduction in the strength of E_01_ compared to the following higher energies.

In the case of nanostructured amorphous polymeric films, the band gap $E_{g}^{TL}$, which is calculated using the TL dispersion equation and includes the apparent absorption resulting from disordering and localized defect states [32,33,34,35,36]. To accurately determine the fundamental band gap, which determines the color emission and is associated with the energy difference between the highest occupied molecular orbital (HOMO) and the lowest unoccupied molecular orbital (LUMO), we utilized the Tauc-plot method [35]. This method involves extrapolating the calculating [*E* × *ε*_2_]^1/2^ to the zero ordinate to obtain a relatively wide band gap. The results of the $E_{g}^{Tauc}$ are presented in Table 1 for comparison.

One can see that the PFO and PBEHF exhibit similar values for the first electronic absorption, which could be ascribed to the fluorene unit, whereas the F6PC presents different values of electronic absorption despite containing the fluorene unit in the polymer chain. In addition, the calculated values of the optical band gap $E_{g}^{Tauc}$ for commercial polymers, PFO, PBEHF, and F6PC also present differences. Among the commercial polymers consisting of the main unit fluorene, the F6PC exhibits the higher value of the $E_{g}^{Tauc}$, which is equal to 3.28 eV. We can distinguish that the presence of the carbazole moiety affects the value of the band gap $E_{g}^{Tauc}$ of F6PC, as it presents a higher value. This can be explained by the shortening of the conjugation length as the carbazole unit inserts into the conjugated backbone of PF, leading to an increase in the energy gap [9,37]. On the other hand, the two values of the band gap of CzCop present lower energies compared to the other ones and more electronic absorptions are observed with significantly broader characteristics. Specifically, the calculated $E_{g}^{Tauc}$ has the lowest value compared to the other three commercially supplied polymers and is calculated equal to 2.67 eV. This may be related to disorder-induced nanostructure and the formation of localized defect states in this film [33].

### 3.2. Absorption Coefficient and Photoluminescence

**Figure 3:**
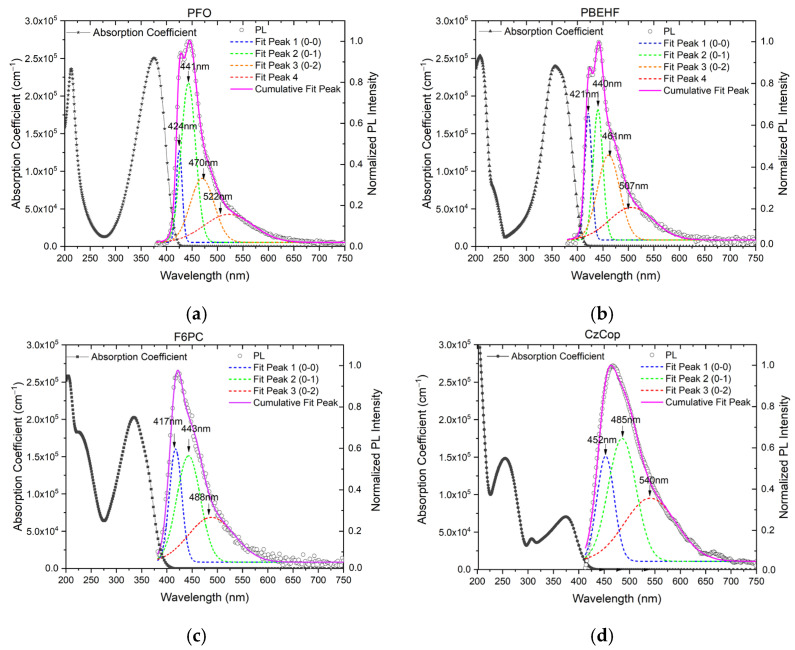
The absorption coefficient and PL emission spectra of (**a**) PFO, (**b**) PBEHF, (**c**) F6PC, and (**d**) CzCop.

The Absorption Coefficient and PL spectra of thin films of PFO, PBEHF, F6PC, and CzCop are presented in Figure 3a, b, c, and d, respectively. The absorption coefficient spectrum of each emitting polymer is derived using the calculated bulk $\varepsilon \left(\omega \right)$. The spectra of the studied polymers show absorptions from 420 to 200 nm. In the case of the absorption coefficient spectrum of PFO, the dominant peak is located at 370 nm, which is attributed to the π-π* transition of the conjugated PF backbone [6,23]. The absorption coefficient spectrum of PBEHF shows a dominant maximum peak at around 360 nm, which is ascribed to π-π* transition [26,29]. The other PFO derivative containing the carbazole moiety, namely F6PC, exhibits a slightly blue-shifted absorption peak at 335 nm in comparison to PBEHF and PFO. This blue shift of the absorption peak can be attributed to a π-π* transition related to the presence of the carbazole unit. Sergent et al. presented the study of photophysical properties of blue-emitting fluorene-co-carbazole-based polymers, and they observed the blue shift of the absorption when the carbazole unit was incorporated along with fluorene [8]. They also proposed that this phenomenon is due to the interruption of the conjugation by the presence of 3,6 carbazole units within the conjugated main chain [8,37,38]. In the case of the novel copolymer CzCop, it is clear that the absorption spectrum shows a broad structureless band from 425 to 300 nm, centered at 380 nm, indicating that it exists in the disordered (amorphous) phase.

Moreover, the emitting thin films were also examined by PL in order to define the luminescence behavior of the active materials. The PL spectra were recorded upon excitation at 370 nm. The right axes of Figure 3a, b, c, and d depict the PL spectra of PFO, PBEHF, F6PC, and CzCop, respectively. For a better evaluation of the PL emission spectra of the emitting films, a deconvolution fitting analysis of the experimental spectra was realized using a Gauss oscillator for the analysis procedure. The deconvolution analysis revealed that the PFO PL spectrum exhibits a vibronic structure with peaks at ~424 nm (0–0), ~441 nm (0–1), and ~470 nm (0–2); a fourth phonon side band (0–3) can also be seen at ~522 nm [6,39]. The PL spectrum of PBEHF is dominated by four distinct peaks, and notably, the PBEHF spectrum is typical of polyfluorenes, similar to the PL spectra of PFO. In particular, it shows a structured band with two sharp peaks at ~421 and ~440 nm, one at ~461 nm, and a minor shoulder at ~507 nm. Therefore, as mentioned above, the first three peaks correspond to an average vibronic progression of the 0–0, 0–1, and 0–2 intrachain singlet transitions, respectively [8,26,29,40,41]. On the other hand, it is remarkable to observe that the shape of the F6PC PL spectrum is different compared to the PFO and PBEHF. For the F6PC, the featureless PL emission was deconvoluted by three peaks located at ~417, ~443, and ~488 nm. The deconvolution analysis reveals a slight blue shift, especially from 421 or 424 to 417 nm. This fact makes us speculate that the carbazole unit affects the emission spectra of F6PC, as the emission is mostly governed by a radiative decay from the electronic states of the conjugated polymer comprising the carbazole unit [8,37,38]. This is due to the interruption of the delocalization of the π-electrons along the polymer backbone by the 3,6-carbazole linkages. Thus, as mentioned above, the presence of the carbazole unit indicates that the conjugation length is reduced, leading to an increased energy gap and resulting in a blue shift in PL emission. In addition, the PL emission of the CzCop presents a similar band shape compared to the F6PC PL spectra, as both F6PC and CzCop contain a carbazole unit in the main backbone. The spectrum of CzCop is shifted to longer wavelengths compared to the F6PC spectrum, as it is also indicated by the deconvolution analysis. This fact is assigned to conjugation disruption because of the ether linkages and structural disorders. The polyether backbone induces non-planar conformations that lead to more electronic transitions. Also, the electron-withdrawing/donating nature of the sulfone group and the Cz and Py groups could lead to more Charge Transfer states.

**Figure 4:**
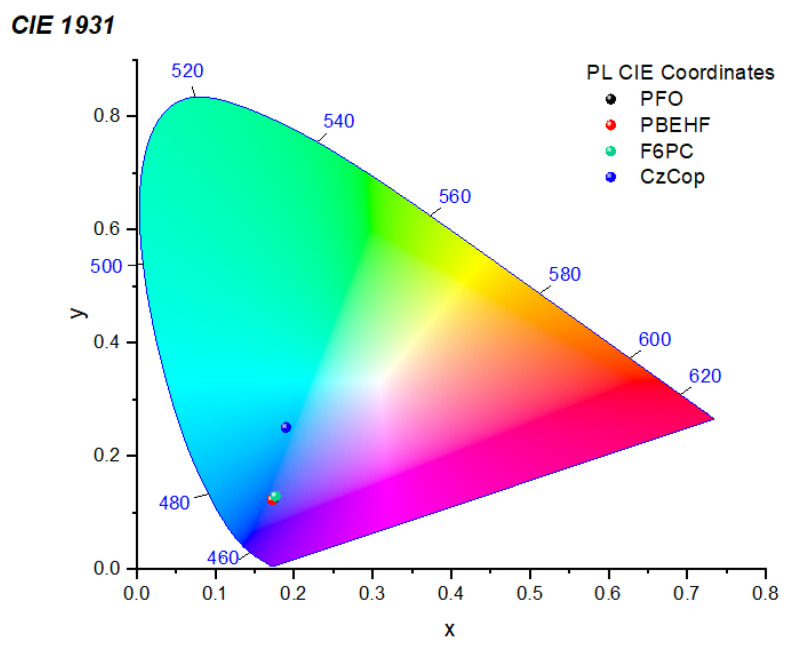
CIE diagram of PL emission of studied emitting materials.

At the same time, the PL measurements are used to calculate the coordinates in the Commission Internationale de L’ Eclairage (CIE) chromaticity diagram, as they are demonstrated in Figure 4. One can observe that the chromaticity coordinates are generally located in the spectral region of the blue region. More specifically, commercially available photoactive materials have coordinates situated in a region characterized by blue emission color [17]. For the PFO, PBEHF, and F6PC, the values of the corresponding coordinates are (0.17, 0.13), (0.17, 0.12), and (0.17, 0.13), respectively. On the other hand, concerning the synthesized polymer CzCop, its CIE Coordinates deviate from the deep blue region, and the emission can be characterized as sky-blue [17]. More specifically, the values of the CzCop chromatic coordinates are (0.19, 0.25).

### 3.3. Atomic Force Microscopy

**Figure 5:**
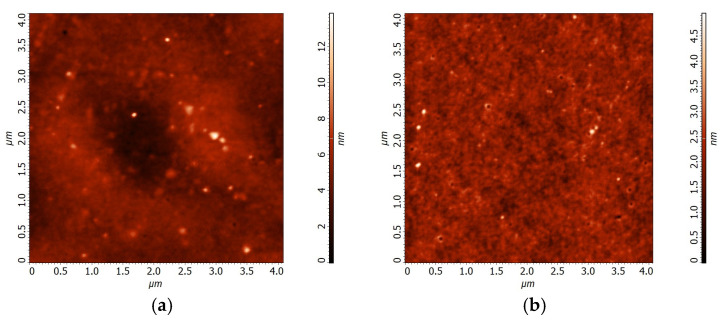

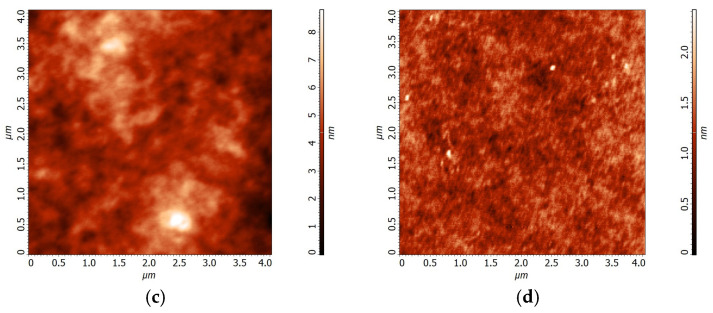
AFM topography Images Scan size 4 × 4 μm of (**a**) PFO, (**b**) PBEHF, (**c**) F6PC, and (**d**) CzCop.

**Figure 6:**
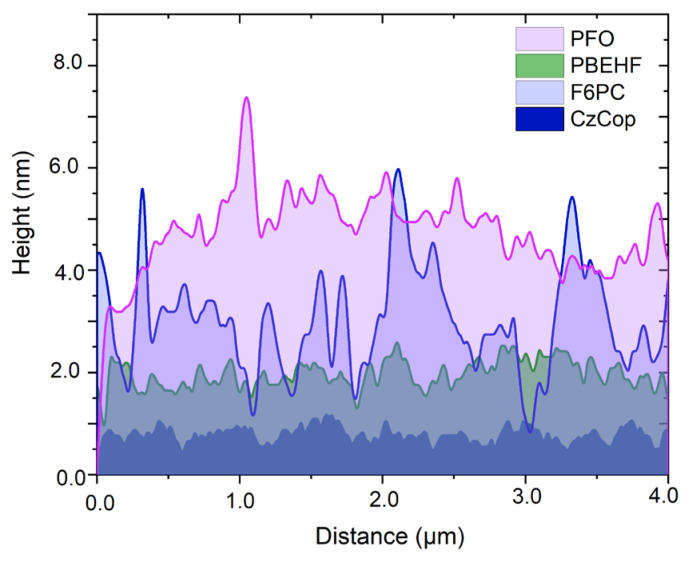
Cross section of AFM images of examined emissive thin films.

Understanding interfaces is crucial for the further development and optimization of OLED devices as they encompass several thin film layers. In particular, the film morphology of the emissive layers is an important parameter that could be taken into account when introducing interfacial layers via wet techniques in optoelectronic devices. For this reason, we extensively studied the surface topography of the emissive thin films using AFM. Figure 5a, b, c, and d illustrate the AFM-measured surface topography (height) images of spin-coated thin films PFO, PBEHF, F6PC, and CzCop, respectively. The results derived from the AFM image analysis are presented in Table 2. It can be observed that the surface morphology of every sample was uniform and sufficiently covered the substrate. The image analysis revealed that almost smooth and continuous films were formed with low Root Mean Square (RMS) roughness values. More specifically, from the height distribution plots shown in Figure 6, it can be seen that most of the features detected are between 2 and 7 nm in height. However, it is noteworthy that the synthesized emitting polymer CzCop thin film exhibits the lowest RMS value compared to the other commercial polymers. The importance of this topic is related to the fact that the interfacial area between the thin films sets the condition for the injection of charges in a device, which has a profound influence on the device’s operational characteristics, for example, the current-voltage characteristic. Generally, it has been established that smoother surfaces reduce the loss of the injection and transportation of charges at the interface, which is beneficial in OLED devices consisting of different layers [13,42]. Thus, the roughness of the emissive layer plays a significant role in the performance of the device and improves the charge injection in the optoelectronic devices, resulting in reduced turn-on voltages.

### 3.4. Electroluminescence

**Figure 7:**
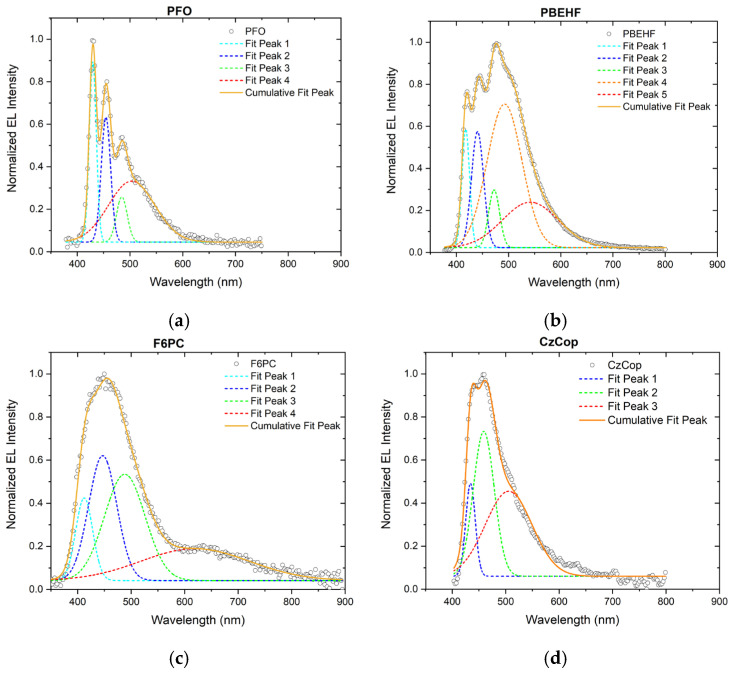
Deconvoluted normalized EL curves of (**a**) PFO, (**b**) PBEHF, (**c**) F6PC, and (**d**) CzCop.

Electroluminescence preliminary investigations on the studied emissive materials were also carried out in order to evaluate their potentiality for the OLED technology. The EL spectra were recorded in the wavelength range from 380 to 900 nm by applying an external bias voltage from 3 to 14 V with 1 V step. In the case of CzCop, the maximum applied voltage was limited to 9 V due to the thinner active layer. Figure 7 shows the respective experimental EL spectra of the studied devices, which were recorded at 14 V for the commercial materials and at 8 V for the lab-scale synthesized polymer. The corresponding theoretical curves were obtained after the fitting deconvolution procedure using 4 Gauss oscillators for the PFO and F6PC, 5 Gauss oscillators for the PBEHF, and 3 Gauss oscillators for the CzCop. Table 3 summarizes the PL and EL emission spectra analysis, including the wavelengths of the λ^max^ peaks and the broadening FWHM.

**Figure 8:**
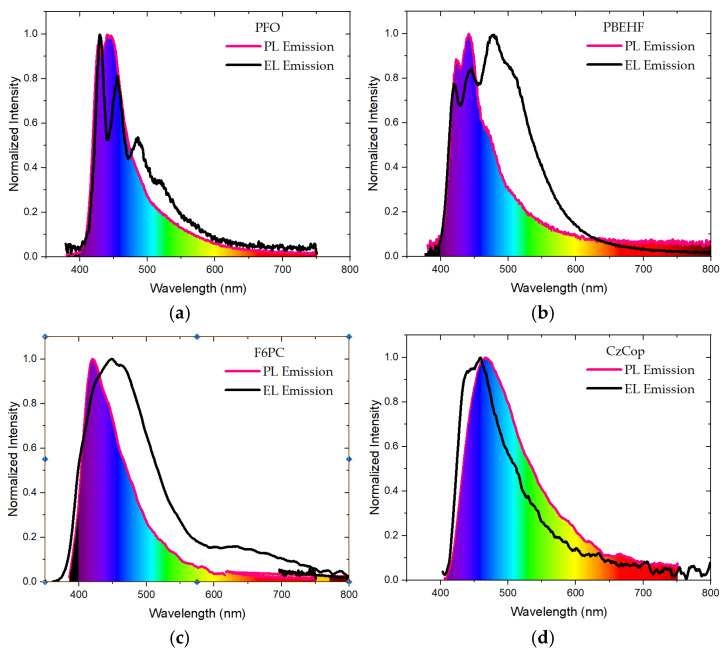
Comparison between EL and PL emission spectra of (**a**) PFO, (**b**) PBEHF, (**c**) F6PC, and (**d**) CzCop.

The comparative study between the Electroluminescence and Photoluminescence spectra is shown in Figure 8 to better comprehend the emission characteristics. For the commercial light-emitting polymers PFO, PBEHF, and F6PC, it is obvious that the emission bands obtained from these studied devices are slightly broadened compared to their corresponding PL spectra. One can see that the maximum EL emission peaks of these commercial polymers are also moved to the longer wavelength region compared to their PL spectra. Specifically, from the EL spectrum of PFO can be observed a low-energy emission peak, which is located at approximately ~500 nm. The EL emission of PBEHF exhibits the dominant peak located at ~500 nm. As previously mentioned, the origin of the green-color emission in PF-type materials has been extensively debated in recent years. There are two possible explanations for this phenomenon. The first one is that the formation of a low-energy emission band at 2.2–2.3 eV occurred due to the formation of fluorenone defect sites (keto-defect) during the device operation, specifically in atmospheric conditions. In fact, it was shown that keto-defects form easily with the oxidation of monoalkylated fluorene monomer units during the device operation [23,24,25,26,43]. List et al. [24] presented in their study that the keto defects act as low-energy trapping sites for singlet excitons, being populated by an excitation energy transfer from the PF main chain. In particular, they found a much stronger contribution from the defect-related emission in EL than in PL spectra, which was attributed to two parallel processes: trapping of charges at the keto site and their subsequent emissive recombination in addition to energy transfer of singlet excitons from the PF main chain to keto defect sites. The other possible explanation is based on the aggregations and/or excimer formation in these materials, originating from the interchain attraction in the π-conjugated systems [44]. Since such interactions are short-range forces, the distance between the polymer chains is one of the governing factors for this phenomenon. As they become smaller, the polymer chains have a higher chance of entanglement with each other to form aggregates.

In the case of F6PC EL spectra, we can observe a dominant peak approximately at 455 nm and a shoulder at 650 nm. The latter probably originated from an electric-field-induced electromer emission. Deksnys et al. [45] observed EL emission in the long-wavelength region due to the electromer emission. In their study, they presented the synthesis, spectroscopic, thermal and electrochemical characterization of the ambipolar fluorophore 3,6-di(4,4′-dimethoxydipheny laminyl)-9-(1-naphthyl) carbazole (DPNC) which demonstrated a voltage-dependent green–blue electrofluorescence. It is well known that the electromer emission takes place mainly from the direct irradiative recombination of holes and electrons residing at two neighbouring molecules or from two molecules within some appropriate close distance.

Finally, deconvolution analysis verified that the novel emitting polymer CzCop has similar emission spectra for both EL and PL. However, we can observe the blue shift in EL spectra compared to PL because PL and EL mechanisms are different. It is well known that the PL emission is associated with the direct photoexcitation of the emissive thin film and the recombination from the excited states, whereas the EL depends on the carrier injection mechanisms, the transportation, and the recombination of charges across the device structure [32,36]. So, when comparing the PL and EL emission spectra of each emitting polymer, it is obvious that the novel lab-scale polymer CzCop exhibits the highest stability and color selectivity in its emission either as a photoactive thin film or as the active layer of an OLED device.

**Figure 9:**
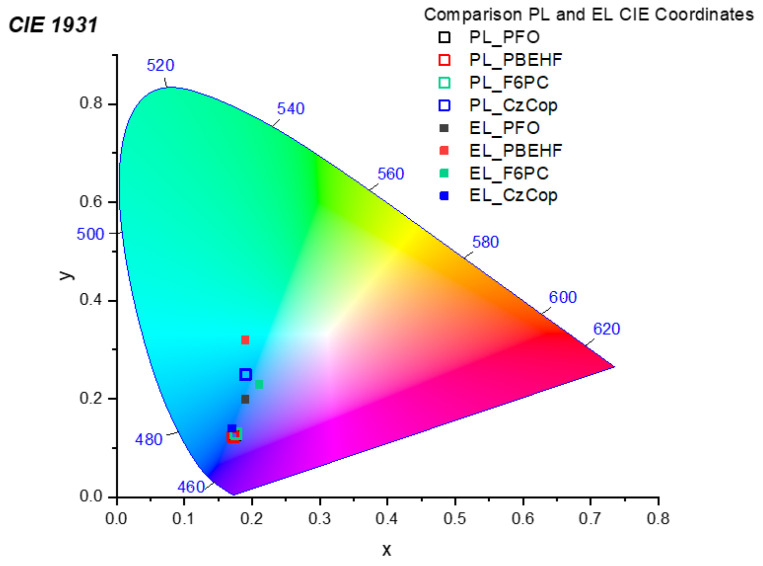
CIE diagram of PL and EL emission of the studied emitting films.

The CIE coordinates, derived from PL and EL measurements for the studied OLEDs, are illustrated in Figure 9. Generally, the CIE coordinates visualize the entire range of colors that can be obtained by mixing the three primary colors (red (R), green (G), and blue (B)) by varying the wavelength and emission intensity. According to the CIE diagram, it is obvious that the PL emission of commercial materials is approaching the blue region. On the other hand, in the case of lab-scale material, the PL CIE coordinates are located in the sky-blue region. Clearly, the PL CIE coordinates are different from the EL ones. Notably, the red shift of the EL emission spectra of the commercial materials is confirmed with the shift of CIE coordinates to the sky-blue region. As it is referred to above, the EL emission spectra exhibit different emission behavior compared to the PL. Specifically, the blue emission of the polymers turns into blue-green emission, as verified through the CIE diagram, except for the lab-scale material. The light of the CzCop device well approaches the blue region, making it a promising candidate as an efficient blue light-emitting material.

**Figure 10:**
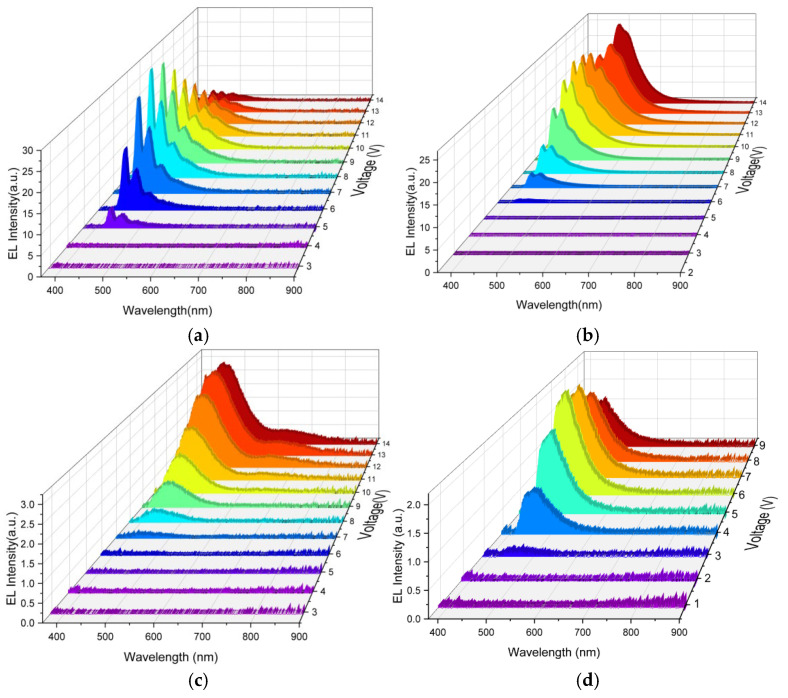
The evolution of EL emission spectra of the produced OLED devices, bearing as active film (**a**) PFO, (**b**) PBEHF, (**c**) F6PC, and (**d**) CzCop, under excitation with various bias voltages.

EL emission under different applied voltages was recorded to assess the crucial stability factor during OLED device operation. Figure 10a–d show the results obtained from the PFO, PBEHF, F6PC, and CzCop devices accordingly. Firstly, comparing the EL spectra of PFO for different applied voltages (1–14 V), an EL peak appeared at approximately ~500 nm, which increases with the applied voltage. Note that the additional featureless green emission may originate from the fluorenone moieties. This can be explained by the fact that the blue light originates from the bimolecular recombination of free electrons and holes, whereas the green light is generated by electrons that are trapped at fluorenone sites and subsequently recombine with a hole. So, the EL spectra of PFO are affected by the defects during the device operation.

In addition, it can be seen that increases in the applied voltage (1–14 V) also induced changes to the EL spectral shape of PBEHF. At higher operation voltages, we can observe EL spectra with multiple peaks that span from the blue to the red region. The ratio of the intensity at 425 nm to 476 nm increased when the voltage increased from 7 to 12 V. When the applied voltage exceeded 12 V, another dominant peak at 507 nm emerged. As mentioned above, the presence of a peak at 507 nm is related to the defects of fluorene. More specifically, it is noteworthy that the green peak presents lower intensity relative to the blue peak, from 7 to 12 V. We can also assume that the blue light originates from the bimolecular recombination of free electrons and holes, whereas the green light is generated by charges that are trapped at the fluorenone defects and subsequently recombine. As a result, the blue emission will strongly increase with voltage, whereas the recombination from the trapping sites is limited by the number of traps [23]. However, when the applied voltage is increased, the intensity of a peak at 507 nm is increased as well. This can be explained by the fact that more charges are trapped at the fluorenone units due to the electrooxidation during the device operation.

For the case of the F6PC device (Figure 10c) and the respective EL spectra obtained under different applied voltages (1–14 V), it is obvious that when the applied voltage is increased from 7 to 10 V, the intensity of a dominant peak, centered at ~450 nm, is increased as well. When the voltage is increased above 10 V, the intensity of the shoulder emission peak located at 650 nm is also increased. This fact indicates that the electromer emission band demonstrates a clear dependence on the applied voltage. As the electromer-type excited states can only be formed under electrical excitation, their emission is visible only in the EL spectra.

Finally, the evolution of EL emission spectra of CzCop with various bias voltages (1–9 V) was also investigated (Figure 10d). It was derived that the EL spectra were nearly unchanged by increasing the driving voltages, indicating the high stability of CzCop in the EL process either as a luminophor or as the host material. This demonstrates that the newly synthesized material forms uniform coatings without defects that could create traps and, by extension, instability in the emission characteristics of the OLED devices to which they are applied. Thus, CzCop exhibits superior EL stability and emission in the blue region, according to the CIE diagram, compared to the other commercially available materials.

**Figure 11:**
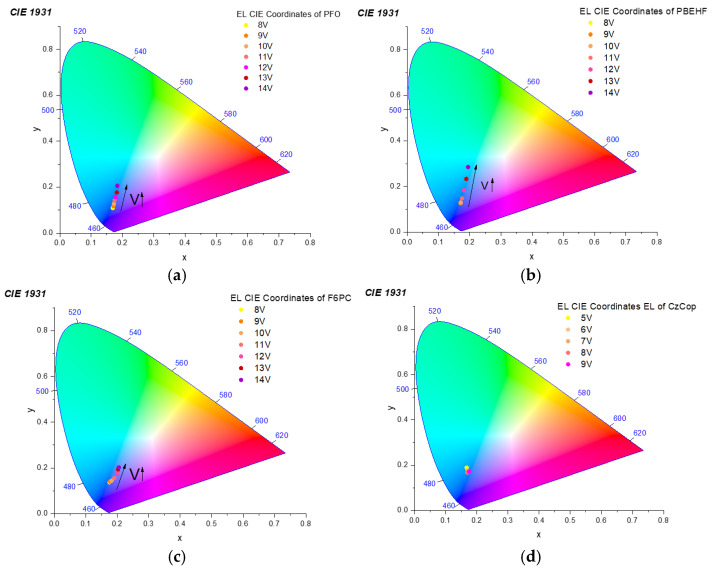
The evolution of EL Chromaticity Coordinates of the produced OLED devices, bearing as active film (**a**) PFO, (**b**) PBEHF, (**c**) F6PC, and (**d**) CzCop, under excitation with various bias voltages. Arrows show the shift of color coordinates towards the sky-blue region with increasing Voltage (V).

The EL spectra of PFO, PBEHF, F6PC, and CzCop OLEDs are converted to the CIE coordinate and overlaid onto the approximate color regions on a CIE 1931 (x, y) chromaticity diagram. The evolution of EL chromaticity coordinates as a function of the applied voltage is illustrated in Figure 11a–d for each polymer. According to the CIE diagram, the emissions from PFO and PBEHF OLEDs were at the edge of the blue-violet region at 8 V, but as the voltage increased, both were emitted to the greenish-blue region. This behavior can be explained as the resultant emission shifted when the intensity of the approximately ~490 nm peak increased. Quite interestingly, the EL chromaticity coordinates of PBEHF present a higher shift compared to the PFO, and this fact can be attributed to the peak emission at ~490 nm, which is the dominant peak at the EL emission, as shown in Figure 11b. For the device based on F6PC, the emissions in the blue area shift towards the center region with increasing voltage, largely due to the appearance of the red emission at 600 nm. Notably, as the applied voltage increases, the novel material CzCop exhibits superior emission stability, independent of the driving voltage. Therefore, the lab-scale polymer CzCop demonstrates excellent stability and color selectivity during the device operation, making it a promising candidate as an efficient blue light-emitting material.

### 3.5. Electrical Characteristics

**Figure 12:**
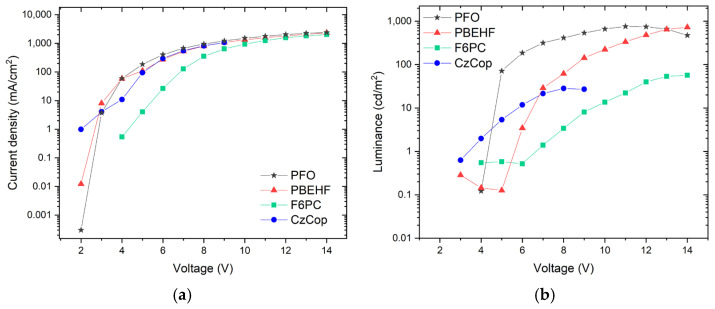
Logarithmic plot of (**a**) Current density–Voltage (J–V) and (**b**) Luminance–Voltage (L–V) characteristic curves of the fabricated OLED devices.

In terms of electrical characteristics, Figure 12a and b show the current–voltage and luminance–voltage characteristics of the fabricated devices on a logarithmic scale, respectively. The electrical characteristics and chromaticity coordinates, which were derived from the EL spectra, are summarized in Table 4. One can see that the novel polymer CzCop exhibits a lower turn-on voltage and lower potential operating voltage compared to the other commercially supplied polymers. It is also important to observe that the novel lab-scale material approaches the electrical characteristics of the commercially supplied ones. The measured luminance at 8 V for the PFO, PBEHF, F6PC, and CzCop is 413, 62, 3, and 28 cd/m^2^, respectively. The maximum luminance measured for the commercial polymers at higher bias voltages exhibits higher values, but this can be ascribed to the fact that their thicknesses are higher compared to the lab-scale polymer CzCop.

Nevertheless, a more extensive and in-depth optimization of the device architecture is essential to enhance overall efficiency. Since it is a novel material, additional device architecture optimization is required in terms of the precise selection and alignment of the Hole Transport Layer-Hole Injection Layer and Electron Injection Layer-Electron Transport Layer [46]. The identification of the most suitable functional materials for each layer in the OLED architecture, along with the smooth surface of the CzCop film that enhances interfacial properties, is expected to result in the production of more efficient devices. Overall, this comparison demonstrates that the commercial materials exhibited undesired blue-green emissions, while the CzCop synthetic polymer nicely supports color stability and selectivity in the pure blue region during device operation, thus paving the way for use in the emerging field of OLEDs. Thus, the CzCop offers promising potential for applications, including displays and medical devices. For the latter, “blue light therapy” has a range of beneficial effects [47].

## 4. Conclusions

In this study, we investigated the optical properties of four different blue light-emitting polymeric materials. Three of these materials (PFO, PBEHF, and F6PC) were commercially available, while the fourth one (CzCop) was a novel polymer synthesized in the laboratory. These materials have been applied as emissive layers in the wet fabrication of OLED devices using the spin coating process. In terms of the optical characterization, a thorough comparison between these materials concerning their thickness, dielectric function, fundamental energy gap, and absorption coefficient was obtained using Spectroscopic Ellipsometry. In addition, quantitative analysis of the PL and EL emission peaks and widths was performed for all the studied materials to evaluate their color stability and selectivity. It was realized that the lab-scale polymer CzCop exhibits superior color stability and selectivity during the device operation. On the other hand, the commercially available PFO, PBEHF, and F6PC show variations in their maximum peak emission wavelength and red shift in their EL spectra. Thus, CzCop is a promising candidate as a blue light-emitting material compared to other commercial polymers. It has great potential for achieving a stable blue color in various high-emergence applications. However, further investigation is required to enhance the functionalization of the fabricated WOLED devices in order to achieve greater efficiency.

## Figures and Tables

**Table 1:** The calculated best-fit results were derived by the analysis of the measured $<\tilde{\varepsilon }\left(E\right)>$ spectra of the films.

	Thickness (nm)	Optical Band Gap (eV)		Electronic Transition Energy (eV)				
		$E_{g}^{TL}$	$E_{g}^{Tauc}$	E_01_	E_02_	E_03_	E_04_	E_05_
PFO	47.0 ± 1	2.87	3.06	3.12			5.54	5.78
PBEHF	42.0 ± 1	2.95	3.11	3.16			4.73	5.66
F6PC	46.0 ± 1	2.99	3.28	3.36	3.50	4.47	5.69	
CzCop	27.0 ± 1	2.67	2.97	3.08	4.02	4.53	5.29	6.18

**Table 2:** AFM results for the spin-coated thin films.

	Root Mean Square, Sq (nm)	Average Roughness, Sa (nm)	Peak to Peak, Sy (nm)
PFO	1.01	0.74	13.72
F6PC	0.86	0.67	8.67
PBEHF	0.32	0.25	4.94
CzCop	0.25	0.20	1.54

**Table 3:** Results of the deconvolution analysis of the PL and EL emission spectra.

	Spectrum	PEAK 1		PEAK 2		PEAK 3		PEAK 4		PEAK 5	
		λ^max^ (nm)	FWHM (nm)	λ^max^ (nm)	FWHM (nm)	λ^max^ (nm)	FWHM (nm)	λ^max^ (nm)	FWHM (nm)	λ^max^ (nm)	FWHM (nm)
PFO	PL	424	12	441	28	470	47	522	96	-	-
	EL	429	13	454	18	484	20	503	88	-	-
PBEHF	PL	421	13	440	19	461	46	507	95	-	-
	EL	418	14	440	23	472	21	492	67	542	107
F6PC	PL	417	24	443	47	488	95	-	-	-	-
	EL	412	32	447	53	488	76	619	20	-	-
CzCop	PL	453	35	488	58	551	113	-	-	-	-
	EL	434	17	458	39	505	88	-	-	-	-

**Table 4:** The electrical–operational characteristics and EL CIE coordinates of fabricated OLED devices.

	Current Density Turn-On Voltage (V)	Luminance Turn-On Voltage (V)	Luminance at 8 V (cd/m^2^)	Luminance (cd/m^2^)	CIEx	CIEy
PFO	2.7	4.0	413	759	0.18	0.20
PBEHF	2.8	5.6	62	729	0.19	0.32
F6PC	4.2	6.6	3	57	0.21	0.23
CzCop	2.0	3.4	28	28	0.16	0.16

## Data Availability

Data presented in this article is available on request from the corresponding author.
